# Flavonoids: Potential Novel Inhibitors of *Mycobacterium tuberculosis*

**DOI:** 10.2174/0118715265361578250504110100

**Published:** 2025-07-03

**Authors:** Kakudji Kisimba, Kabange Kasumbwe, Frederick Odun-Ayo, Mbuso Faya

**Affiliations:** 1 Department of Pharmaceutical Sciences, University of Kwazulu-Natal, Private Bag X54001, Durban 4000, South Africa;; 2 Department of Biotechnology and Food Sciences, Durban University of Technology, Durban, South Africa;; 3 Department of Biotechnology and Consumer Sciences, Cape Peninsula University of Technology, Cape Town 7530, South Africa

**Keywords:** ACC 2, metabolic syndrome, *Mycobacterium tuberculosis*, QSAR, log P, molecular docking

## Abstract

Tuberculosis (TB) is a major global health concern and a leading cause of death worldwide. The emergence of drug-resistant TB strains poses a significant threat to public health and is contributing to the growing rate of TB infections globally. Therefore, it is crucial to explore new and safe drugs for TB treatment. Despite significant progress in developing new drugs, many existing treatments and prevention strategies for TB do not achieve the desired positive health outcomes for various reasons. Small-molecule treatments can potentially address drug resistance and provide opportunities for multimodal therapy. This review focuses on recent advancements in understanding the pathogenesis of *Mycobacterium tuberculosis* and the mechanisms of flavonoids in antimycobacterial properties. Given the urgent need for new antimycobacterial agents to enhance the effectiveness of current drugs, investigating flavonoids as potential candidates is promising. Evidence suggests that specific structural characteristics in flavonoids play a significant role in their antimycobacterial effects, among other pharmacological activities. Flavonoids can act through various mechanisms, such as disrupting bacterial cell membranes or inhibiting the production of essential cellular components like DNA. These findings may prompt further research to enhance our understanding of how flavonoids combat tuberculosis, potentially establishing their importance as key compounds in treating the disease.

## 1 INTRODUCTION

Tuberculosis (TB) is a serious infectious disease caused by the bacterium *Mycobacterium tuberculosis* (Mtb). It can affect various parts of the body, including the skin, lymph nodes, brain, and lungs, and is characterized by the formation of tubercle lesions [1]. Particles from an infected individual can transmit the disease. *Mycobacterium tuberculosis* initially induces a respiratory infection and triggers an immune response upon invading the lung [2]. According to the most recent World Health Organization Global Tuberculosis Report (2023), TB was the second-deadliest infectious disease worldwide in 2022, causing nearly twice as many deaths as HIV/AIDS and resulting in 7.5 million TB diagnoses globally. Isoniazid (INH), pyrazinamide (PZA), rifampicin (RIF), and ethambutol (EMB) are four effective oral antimicrobial drugs used in the treatment of this illness. INH and RIF are prescribed for the first two months, followed by continuing treatment for an additional four to seven months [3, 4]. INH's mechanism of action is intricate despite its basic chemical structure. It targets multiple pathways involved in macromolecular synthesis, focusing on inhibiting mycolic acid synthesis. This disruption in mycolic acid production, a crucial component of the mycobacterial cell wall, impedes the growth of mycobacteria [5]. INH prevents the production of mycolic acid. RIF inhibits the process of transcribing bacterial DNA into RNA by binding to the β-subunit of the enzyme DNA-dependent RNA polymerase within bacterial cells. This binding action disrupts protein synthesis, ultimately leading to the death of the bacterial cells [6]. PZA, once converted to pyrazinoic acid, disrupts trans-translation. EMB interferes with arabinosyl transferases, stopping the formation of mycobacterial cell walls. Aminoglycosides, including streptomycin, kanamycin, and amikacin, bind to the ribosomes' 30S subunit, halting protein synthesis. Fluoroquinolones like levofloxacin, moxifloxacin, and gatifloxacin obstruct DNA gyrase and topoisomerase IV, further impeding DNA synthesis [7].

The complete eradication of this disease has been impeded by the emergence of drug-resistant TB, including multidrug-resistant (MDR), TB extensively drug-resistant (XDR) TB, and the more recent drug-resistant TB. INH and RIF are considered to be among the most potent drugs used to treat TB. However, MDR strains of the disease have developed some degree of resistance to these drugs. Furthermore, XDR strains exhibit high levels of resistance not only to the initial TB drugs but also to certain secondary drugs, such as fluoroquinolones and aminoglycosides [8]. The complexity of the disease, the duration of the treatment plan, the value of drug sensitivity testing, and inaccurate diagnosis are all considerable challenges. Furthermore, drugs such as RIF and INH are associated with severe side effects, such as hepatotoxicity [9]. In addition to the complexity of immune system control and disease-causing ability, there has been a worrying increase in the occurrence of drug resistance, specifically 78% MDR in tuberculosis, which has led to decreased chances of patient recovery [10].

New anti-TB drugs that can treat both drug-sensitive TB and drug-resistant TB, including multidrug-resistant TB, are required to manage and eliminate this global health crisis. The increase in tuberculosis cases has led to a need for new drugs that target different areas of the disease [11]. As *M. tuberculosis* relies on its cell wall for survival and virulence, enzymes responsible for cell wall formation are attractive targets to inhibit TB infection. The mycobacterial cell wall's ability to allow substances to pass through and remain intact relies on mycolic acids, which are long-chain fatty acids.

A proven anti-TB drug discovery approach involves targeting the biosynthesis pathway of mycolic acids, as seen in drugs like INH, EMB, and the clinical MmpL3 inhibitor SQ109 [12]. Several new drugs, such as bedaquiline, delamanid, and pretomanid, have been discovered to enhance MDR and XDR outcomes, but they come with notable side effects despite their improved effectiveness and advantages compared to existing first-line tuberculosis treatments [3]. For instance, bedaquiline, a recently introduced drug in clinical use, has been found to negatively impact the heart rate [13]. The development of a new anti-TB drug that reduces side effects and toxicity, effectively targets MDR and XDR, and penetrates host cells is of great importance and would significantly impact public health [14]. Various approaches have been investigated in the search for the ideal drug, such as repurposing existing drugs like clofazimine, designing drugs based on structure and mechanism with the help of genome sequencing, pinpointing molecular targets, and combining active pharmacophores through molecular hybridization [9].

The increasing global prevalence of multidrug-resistant *Mtb* necessitates innovative treatment approaches to improve the effectiveness of existing medications [15]. Natural products and their derivatives have been recognized as a valuable source of bioactive molecules for medicinal purposes since ancient times, predating the development of modern treatments [16]. Extensively studied in the natural products field, flavonoids, a type of polyphenolic compound, are commonly found in a variety of foods like fruits, vegetables, legumes, grains, spices, nuts, and plant-based products such as tea, coffee, and red wine [17-19]. According to Torres-Piedra *et al*. [16], flavonoids are molecules capable of interacting with multiple targets, which is why they are classified as privileged structures. Flavonoids have a broad structure consisting of two phenyl rings connected by a 3-carbon heterocyclic ring, totalling 15 carbons in the compound's skeleton [20]. Based on the changes in the central carbon ring, they can be divided into the following subclasses: flavonols, flavanones, isoflavones, flavones, flavan, and anthocyanidins [21]. Flavonoids, derived from plants and fungi, are extensively studied natural compounds with various therapeutic properties such as antibacterial, antioxidant, anti-inflammatory, anti-cancer, and antiviral effects [22, 23]. Their health benefits have sparked intense research efforts aimed at uncovering additional biological properties and processes. Recently, certain flavonoids have attracted significant attention due to their distinct biological roles in regulating multidrug resistance, as stated by Xiao *et al.* [24]. Currently, the main focus of antibacterial studies is on semisynthetic and synthetic flavonoids due to their higher antimicrobial effectiveness; certain compounds are 16-32 times more potent than natural flavonoids [25].

Molecular docking is an attractive method in the field of drug discovery and design, as it aids in understanding the interaction between drugs and proteins/DNA to form a reliable complex with strong efficacy and specificity. This is very advantageous for discovering new targets for therapy. Due to this approach, molecular docking has become an extremely valuable method for discovering and developing new and promising drugs [26]. Modifying potential lead molecules structurally remains a key strategy in developing new therapeutic drugs. This review presents a comprehensive evaluation of the significance of flavonoids in the context of potential anti-tubercular agents, highlighting their role as lead candidates. It also discusses the potential impact of emerging targets in TB drug discovery in the foreseeable future.

## 2 MATERIALS AND METHODS

The review focused on fully published studies written in English from journals indexed in databases such as Scopus, ScienceDirect, PubMed, and Web of Science. There were no period restrictions, and all articles related to the topic were considered. The search used descriptors such as Tuberculosis, Biology of Tuberculosis, Flavonoids, Chemical Structures, Molecular Targets against *M. tuberculosis*, and Flavonoids against Drug-Resistant Tuberculosis Strains. Initially, articles were pre-selected based on their abstracts, and then the selected articles were read in full. A total of 104 articles were identified during the search. The data were organized as follows: Biology of tuberculosis, chemical structure and subclasses of flavonoids, molecular targets against *M. tuberculosis*, potential molecular targets associated with the discovery of novel ligands, *In silico* studies on the potential inhibitory effects of flavonoids on Mtb proteins, flavonoids against Non-tuberculous Mycobacteria, and flavonoids as a potential solution to combat drug-resistant tuberculosis strains. The process for selecting articles included in the review is outlined in Fig. (**1**).

### 2.1 Inclusion Criteria for Studies in this Review Include

1. Studies investigating the antimycobacterial properties of flavonoids.
2. Studies exploring the mechanisms of action of flavonoids against *Mycobacterium tuberculosis*.
3. Studies focusing on the potential use of flavonoids as antimycobacterial agents.
4. Studies examining the structure-activity relationship of flavonoids in relation to their antimycobacterial effects.
5. *In vitro* studies evaluating the efficacy of flavonoids against *Mycobacterium tuberculosis*.
6. Studies published in English language.

## 3 BIOLOGY OF TUBERCULOSIS

**Fig. (2):**
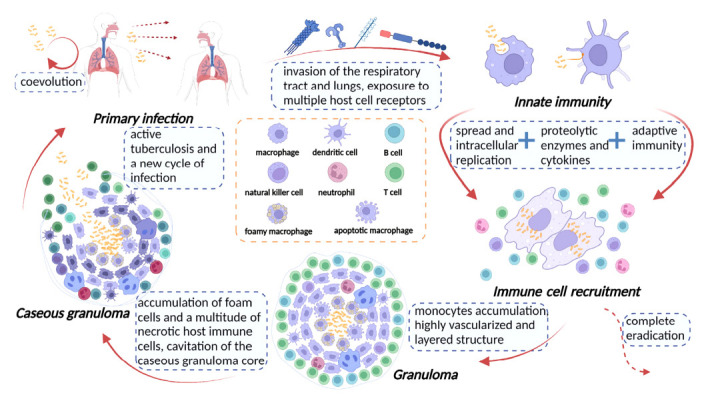
Pathogenesis of tuberculosis.

Tuberculosis is a contagious illness caused by *Mtb,* a highly effective pathogen that primarily affects the lungs and leads to the typical symptoms of pulmonary TB. Additionally, extra-pulmonary TB can affect various organs and tissues, including lymph nodes, brain, kidneys, and spine [27]. The bacteria are transmitted and inhaled through small droplets released by coughing, speaking, singing, and sneezing of contagious individuals [28]. In patients infected with *Mtb*, the sizes of infectious droplets vary from 0.65 (small) to > 7.0 μm (medium-large) [29]. *Mycobacterium tuberculosis*’*s* ability to manipulate host macrophages, form granulomas, and enter a dormant state is essential for its pathogenicity, as it allows the bacteria to resist host defenses and treatment [30]. The majority of droplet nuclei containing *Mtb* from contagious individuals are trapped in the upper airway and expelled by ciliated mucosal cells, with only a small fraction reaching the alveoli. *Mtb* attaches to alveolar macrophages using complement receptors, alters the phagosome environment, and modifies cell wall components [31]. Thus, the transmission of *Mtb* occurs through airborne droplets when an infected person coughs or sneezes, entering the body through the respiratory tract. Once in the lungs, *Mtb* is engulfed by macrophages, where it can survive, replicate, and recruit more immune cells to the infection site. Granulomas form, composed of foam cells and necrotic immune cells, leading to caseous granulomas that may rupture and release bacteria, causing active TB. Infectious aerosol droplets are then released, perpetuating the infection cycle (Fig. **2**) [32].

## 4 CHEMICAL STRUCTURE AND SUBCLASSES OF FLAVONOIDS

### 4.1 Flavonoids

**Scheme 1:**
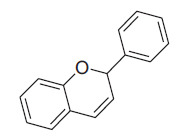
The basic structure of flavonoids.

Flavonoids are a diverse group of phenolic compounds derived from plants and can be found in glycosylated and non-glycosylated forms [33]. Flavonoids are composed of a 15-carbon skeleton as the fundamental structure, with two benzene rings (A and B) linked by a heterocyclic pyrene ring (C) that contains oxygen. Flavonoids that lack the C ring are referred to as chalcones and are frequently used as precursors for other types of flavonoids [34, 35]. These compounds are typically hydroxylated at positions 5 and 7 on the A ring and oxidized at the 3′,4′, or 3′,4′,5′ positions on the B ring as a result of their biosynthetic pathways [36]. The various classes of flavonoids exhibit differences in oxidation levels and substitution patterns of the C ring, with individual compounds within a class showing variations in substitution patterns of the A and B rings (Scheme **1**) [37].

**Scheme 2:**
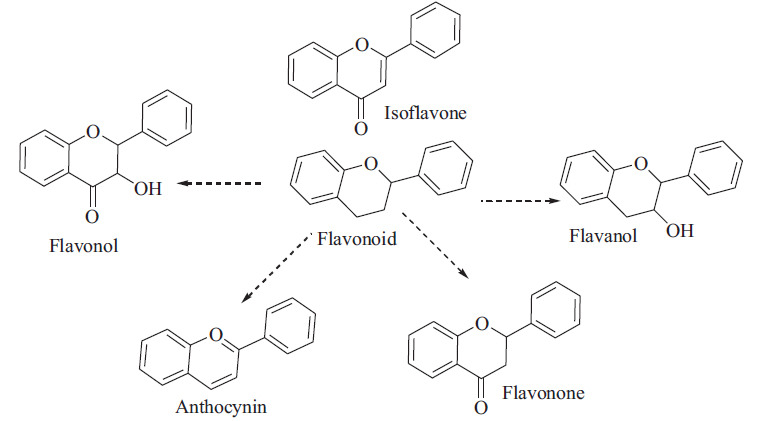
Major classes of flavonoids.

Flavonoids can be classified into different subgroups depending on the attachment position of the B ring on the C ring, as well as the degree of unsaturation and oxidation of the C ring. Isoflavones are flavonoids where the B ring is attached at position 3 of the C ring [38]. Neoflavonoids have the B ring attached at position 4, while those with the B ring attached at position 2 can be further classified into different subgroups based on the structural characteristics of the C ring. These subgroups include flavones, flavonols, flavanones, flavanonols, flavanols or catechins, anthocyanins, and chalcones (Scheme **2**) [39-41].

Table **1** presents various mechanisms through which flavonoids can combat mycobacteria, as described in the literature.

### 4.2 Flavones and Flavonols

**Scheme 3:**
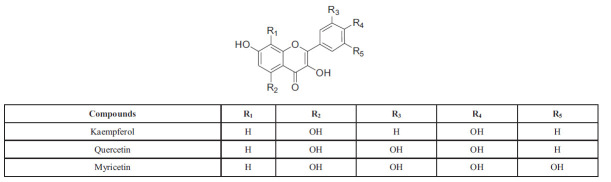
Flavonols: the basic structure, different types, and positions of substitution in the chemical skeleton.

**Scheme 4:**
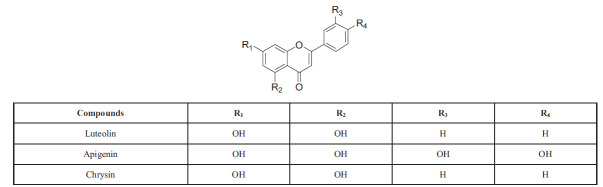
Flavones: chemical structure, various types, and positions of substitution within the core structure.

Flavones and flavonols are important classes of flavonoids. Flavones, also known as 2-aryl-4H-chromen-4-ones, are derived from flavanones through dehydrogenation, while flavonols, or 2-aryl-3-hydroxy-4H-chromen-4-ones, are derived from dihydroflavonols. Flavones are widely distributed and are considered the most representative class of flavonoids, with flavonols being a type of 3-hydroxyflavones. Due to their abundance in nature and known biological activities, flavones have attracted significant scientific interest [49]. Flavones can be further classified based on their substitution patterns and distribution, such as O-methylated, C-methylated, and isoprenylated flavones. Flavonols, in contrast, are key flavonoids present in a variety of fruits and vegetables such as apples, berries, grapes, tomatoes, and onions. They are essential for attracting pollinators and dispersing seeds [50]. Quercetin is one of the most prevalent flavonols with established biological properties and can exist in both aglycone and O-glycoside forms. O-glycosylation typically occurs at C-3, as seen in rutin, a widely distributed flavonol glycoside in the plant kingdom. The subclasses of flavones and flavonols are illustrated in Schemes **3** and **4**, respectively [51].

Flavones, including luteolin, apigenin, and chrysin, are important flavonoids (Scheme **4**) [52]. These compounds are mainly found in leaves, flowers, and fruits as glucosides of apigenin, luteolin, and diosmetin [53].

Yadav *et al*. [54] investigated the antitubercular activity of 15 flavonoids against the *M. tuberculosis* H37Rv strain using the BACTEC 460 assay. Their study revealed five novel antitubercular compounds: luteolin, baicalein, quercetin, myricetin, and hispidulin, with MIC values ranging from 25 to 100 µg/mL.

**Scheme 5:**
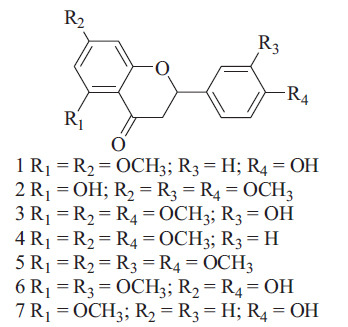
Seven methoxy-flavones were identified and tested against *M. tuberculosis.*

In a study on plant compounds and their impact on mycobacteria's susceptibility to isoniazid (INH), various flavonoids were investigated for their potential to reduce the MICs of INH. The structure-activity relationships of flavonoids such as epicatechin, isorhamnetin, kaempferol, luteolin, myricetin, quercetin, rutin, and taxifolin were analyzed. Among all the strains tested, myricetin exhibited the highest ability to increase susceptibility to INH, particularly in *M. smegmatis* mc2155 and *M. phlei*, where the most significant synergistic effects were observed [55]. Castellar *et al*. [56] isolated and identified active antimycobacterial compounds from *Lippia lacunosa Mart*. & *Schauer*. Seven methoxy-flavones were identified and tested against *M. tuberculosis*. All compounds (Scheme **5**) exhibited antimycobacterial activity against the susceptible strain.

### 4.3 Flavanones

Flavanones are distinct from flavones due to the presence of a single bond between C2 and C3 in the C ring. They are commonly found in the pulp of citrus fruits such as oranges, lemons, and grapes. Hesperidin and naringenin are two well-known flavanones. Hesperidin features hydroxylation and substitution patterns of 5, 7, 4'-OH, while naringenin has 5, 3'-OH, 4'-OMe, and 7-rutinose. The subclasses of flavanones are depicted in Scheme (**6**) [50, 57].

Pawar *et al* [58] used a computational method to evaluate the binding of natural compounds to *Mtb* -MurI. They found that two flavonoids, naringenin, and quercetin, exhibited high binding affinity and effectively inhibited the racemization activity of *Mtb* -MurI. Additionally, these compounds induced structural changes and damage to mycobacterial cells' membranes and cell walls. The findings of this study propose that flavonoids may serve as promising candidates for the treatment of tuberculosis.

### 4.4 Isoflavonoids

Isoflavonoids play a vital role in microbial signaling and nodule induction in legume-rhizobia symbioses. They consist of aglycones, which are the basic structures of isoflavonoids without any sugar molecules attached, and glycosides, which have one or more sugar molecules attached to the aglycone structure. Examples of isoflavonoids include genistin and daidzin, with soybeans being a primary natural source. These compounds are released by legumes as signaling molecules to interact with microbial symbionts. The subclasses of isoflavonoids are illustrated in Scheme (**7**) [59]. These isoflavonoids play a crucial role in facilitating communication between legumes and rhizobia, ultimately leading to the formation of nodules and the establishment of a symbiotic relationship.

**Scheme 8:**
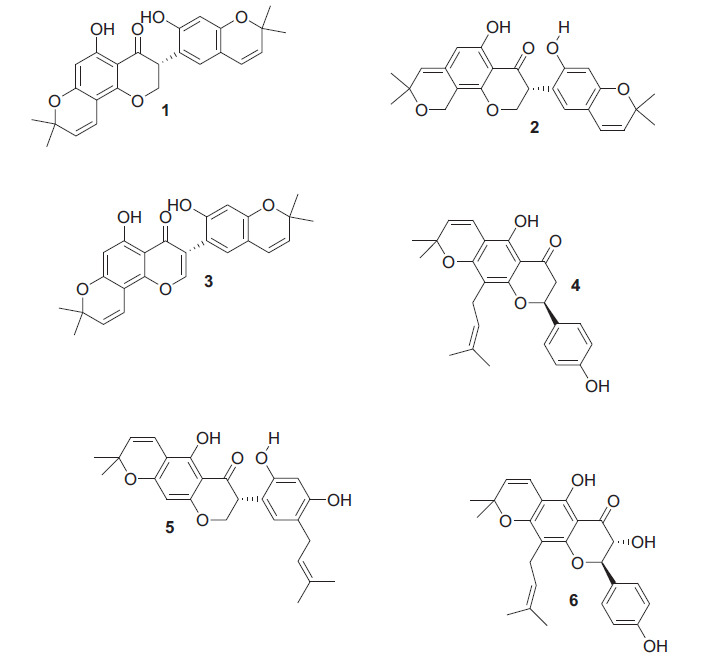
New isoflavonoids extracted from *Rhynchosia precatoria*.

Aceves *et al*. [60] conducted a study on the antimycobacterial properties of new Isoflavonoids extracted from *Rhynchosia precatoria*. The study identified six compounds: precatorin A (1), precatorin B (2), precatorin C (3), lupinifolin (4), cajanone (5), and lupinifolinol (6) (Scheme **8**). Compounds 1 to 5 showed inhibition of *M. tuberculosis* growth at a MIC of 31.25 µg/mL. Compounds 1, 2, 4, and 5 were found to be bactericidal (MBC ≥31.25 µg/mL) and also exhibited inhibitory effects on *M. smegmatis* at MIC of 125 µg/mL. Compounds 1 and 4 demonstrated bactericidal effects (MBC ≥125 µg/mL).

### 4.5 Flavanols

**Scheme 9:**
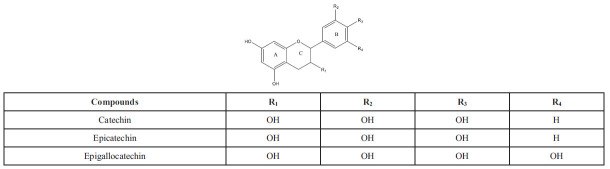
Flavanols: overview of chemical structure, different types, and positions of substitutions within the basic skeleton.

Flavanols are a type of flavonoid, which is a class of plant compounds with antioxidant properties. They are distinguished by the lack of a double bond between C-2 and C-3 and the absence of a carbonyl group on the C-4 ring, along with hydroxyl groups on C-3 or C-4. These compounds can be found in a variety of foods, including cereals, legumes, fruits, vegetables, forages, hops, beers, red wine, tea, cocoa, grapes, and apples. Some well-known examples of flavanols are depicted in Scheme **9** [61]. Flavanols have been studied for their potential health benefits, including their ability to improve heart health, reduce inflammation, and support brain function. Cocoa is particularly rich in flavanols, and dark chocolate is a popular source of these compounds.

### 4.6 Anthocyanidins

**Scheme 10:**
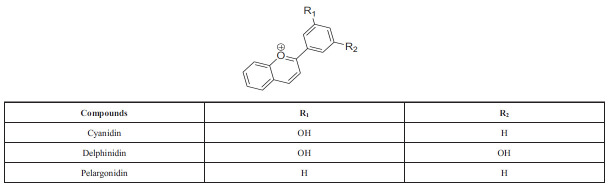
Anthocyanidins: chemical structure, types and substitution positions in the basic skeleton.

Anthocyanins are O-glycosides of anthocyanidins, which are highly oxidized 2-aryl-3-hydroxychromenylium compounds that act as colored pigments. Cyanidin produces red to magenta hues, delphinidin yields purple to blue shades, and pelargonidin contributes to orange to pink tones (Scheme **10**) [50]. These compounds are less stable but are still found in nature, giving color to fruits like apples, grapes, and berries. The specific coloration is determined by the structure of the compound, which can be modified through hydroxylation and methylation at specific positions on the A and B rings, distinguishing them from other flavonoids, except Flavanols [39].

## 5 MOLECULAR TARGETS AGAINST *M. TUBERCULOSIS*

**Fig. (3):**
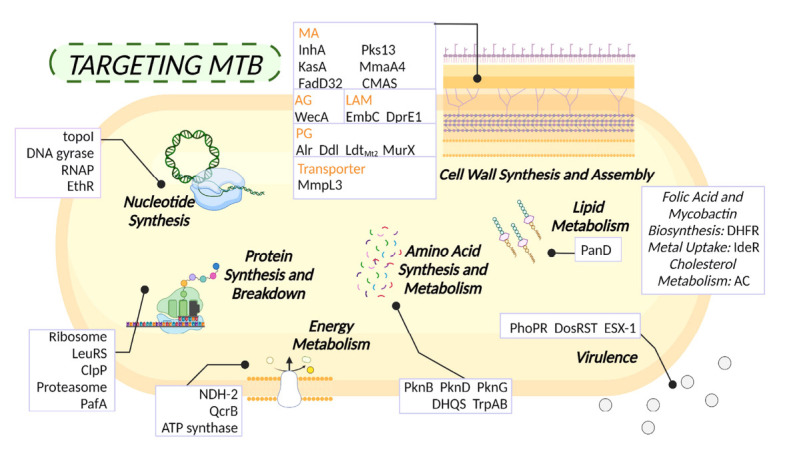
An overview of the targets for addressing tuberculosis in *Mtb* includes disrupting key processes like cell wall synthesis, protein handling, and energy generation. This strategy has been effective in combating the disease.

Scientists are exploring the molecules and biological pathways present in *Mtb* as potential targets for developing new drugs and treatments for tuberculosis, which is caused by this bacterium. The study of the molecular biology and biochemistry of *Mtb* aims to identify key proteins, enzymes, or molecules essential for the pathogen's survival and growth. Blocking or inhibiting these targets with drugs could potentially stop the replication and spread of *M. tuberculosis* in the body. Promising targets include enzymes involved in cell wall synthesis, protein synthesis, respiration, DNA replication, and other critical cellular processes (Fig. **3**) [32]. Identifying and validating these targets is crucial for developing new antibiotics and therapies to combat tuberculosis effectively.

### 5.1 Mycobacterium Cell Wall

**Fig. (4):**
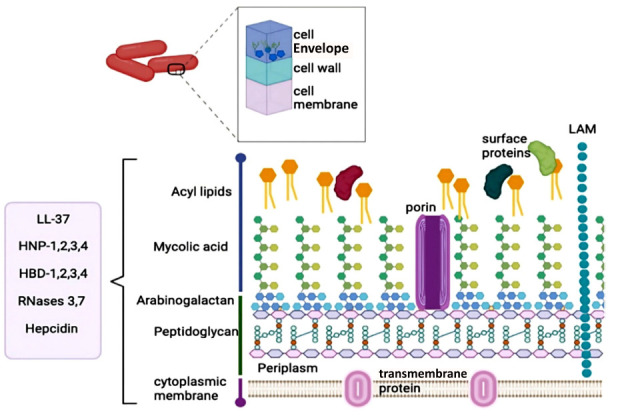
Cell wall of *M. tuberculosis*. The *Mtb* cell wall consists of various intricate layers, such as the cell envelope, cell wall, and cell membrane. Multiple lipids and peptidoglycan make up these layers. The peptides identified to interact with this intricate cell membrane are listed in the box.

The cell envelope of *M. tuberculosis* is known for its dynamic structure, which includes a peptidoglycan layer, a mycolic acid layer, and an arabinogalactan polysaccharide. This complex and unique envelope acts as a protective barrier for *Mtb*, allowing it to adapt to the host environment and evade the immune system. Understanding the composition and function of the cell envelope is crucial for developing effective vaccines and drugs against tuberculosis [62]. The cell envelope consists of the plasma membrane, cell wall, and cell envelope, with around 60% of its composition being complex lipids. The interconnected peptidoglycan layer forms covalent bonds with arabinogalactan, creating a sturdy structure (Fig. **4**) [63]. The mycobacterial cell envelope is made up of five components, including the capsule layer, outer membrane, arabinogalactan, peptidoglycan, and inner membrane [64]. The main structure of the cell wall, mycolyl-arabinogalactan-peptidoglycan, is composed of long-chain mycolic acids, branched arabinogalactan, and cross-linked peptidoglycans [65]. These components play a crucial role in *M. tuberculosis* virulence by disrupting host cell processes [66, 67]. Targeting the cell envelope components, such as with flavonoids, can potentially lead to the development of effective tuberculosis treatments. Flavonoids have been shown to inhibit bacterial growth, disrupt microbial adhesion, and damage bacterial cell walls, making them promising candidates for drug development against tuberculosis [68].

### 5.2 Nucleic acid Synthesis

DNA replication is a biological process that creates an exact duplicate of a DNA molecule. For any organism to survive and reproduce, it is crucial to have accurate copying and upkeep of its genome as part of its total energy consumption [69]. *M. tuberculosis* spreads in humans through a series of cell divisions and passing on of DNA to maintain its position [70]. DNA replication-associated enzyme machinery is extremely crucial for mycobacteria to access nucleotide synthesis, initiation, unwinding, and elongation of DNA in diverse host environments with metabolic, immune, and antibiotic stresses [71]. Therefore, targeting the process of DNA replication is crucial for developing drugs to combat drug-resistant bacteria like *M. tuberculosis*. Numerous proteins essential for these important cellular functions have been utilized for TB drug development. A critical protein target in this area is the mycobacterial DNA gyrase (topoisomerase II), which is produced by the gyrA and gyrB genes. The DNA gyrase catalytic function involves unwinding DNA during replication [72]. Transcription, the initial step in gene expression, involves the synthesis of RNA from a specific segment of DNA by the enzyme RNA polymerase. This process plays a crucial role in regulating the biochemical functions of cells. Bacterial RNA polymerase is a common target for a wide range of antibacterial drugs [73, 74].

Flavonoids are recognized for their ability to inhibit topoisomerases, which play a role in antimycobacterial activity. Topoisomerases are essential for DNA replication and are a primary target for antimycobacterial drugs [75]. Flavonoids inhibit bacterial nucleic acid synthesis by disrupting DNA and RNA synthesis. Quercetin and apigenin block DNA gyrase in Gram-positive bacteria, hindering DNA replication in *Mycobacterium smegmatis* and *Mtb*. Flavonoids bind to DNA gyrase, inhibiting its function and DNA replication [76]. A previous computational analysis study confirmed that quercetin efficiently inhibits DNA topoisomerase at the B subunit of the enzyme, thereby impeding the growth of *M. smegmatis* [77]. Previous studies have shown that quercetin inhibits DNA topoisomerase by competitively interacting with the ATP binding site in the B subunit, causing DNA supercoiling [75, 78, 79].

### 5.3 Protein Synthesis

Protein synthesis, also known as translation, is a vital process in which cells construct new proteins by assembling amino acids at ribosomes. In *Mtb*, the ribosome and leucyl-tRNA synthase (LeuRS) play key roles in protein production. Additionally, the caseinolytic protease P (ClpP) system and the proteasome system are responsible for degrading damaged proteins within the cell [32]. Targeting specific components involved in protein synthesis, such as ribosomes or translation factors, can inhibit bacterial growth and potentially eliminate the bacteria. This strategy is commonly used in the development of antibiotics and other antimicrobial agents to target *M. tuberculosis* and other bacterial pathogens [80].

### 5.4 Energy Metabolism

*M. tuberculosis*, the bacterium responsible for tuberculosis, has several molecular targets that can be exploited for drug development. One important target is the bacterium's energy metabolism. *M. tuberculosis* relies on various metabolic pathways to generate energy for its survival and replication. By targeting key enzymes and proteins involved in these pathways, researchers can develop drugs that disrupt the bacterium's energy production, leading to its death [81]. Some potential molecular targets in *M*. *tuberculosis* energy metabolism include enzymes involved in glycolysis, the tricarboxylic acid (TCA) cycle, and oxidative phosphorylation. For example, inhibitors of enzymes like pyruvate kinase, isocitrate dehydrogenase, and ATP synthase have shown promise as potential anti-tuberculosis agents [82]. Targeting *M. tuberculosis* energy metabolism is a promising approach for developing new drugs to combat tuberculosis, especially in the face of increasing drug resistance. By disrupting the bacterium's ability to generate energy, these drugs can effectively kill *M. tuberculosis* and help in the treatment of tuberculosis infections.

## 6 POTENTIAL MOLECULAR TARGETS ASSOCIATED WITH THE DISCOVERY OF NOVEL LIGANDS

### 6.1 InhA, enoyl-acyl Carrier Protein Reductase

Antimycobacterial drugs target specific enzymes or proteins in *M. tuberculosis*, the bacterium that causes tuberculosis. One such target is InhA, an enzyme called enoyl-acyl carrier protein reductase involved in fatty acid synthesis, specifically mycolic acid biosynthesis, which are essential components of the mycobacterial cell wall [83]. InhA belongs to the Tyrosine-dependent oxidoreductase family, also known as short dehydrogenase/reductase, and is closely related to NADH-dependent enoyl ACP reductase. It speeds up the reduction of trans double bonds by utilizing a carbonyl group in an intermediate that is covalently attached to an acyl carrier protein in the FAS-II pathway [84]. Consequently, InhA is crucial for the growth of M. TB and is a promising target for the development of new antitubercular drugs [85]. InhA inhibitors typically interact with the enzyme's cofactor (NADH), tyrosine amino acid, and lipophilic binding sites, regardless of their structural diversity [86]. Inhibiting InhA disrupts the cell wall synthesis and leads to the death of the bacteria. This makes InhA a key target for antimycobacterial drugs. The binding site of InhA has three key regions: the catalytic site (Site I), Site II, and Site III. Inhibitors interact with InhA through hydrogen bonds, hydrophobic interactions, and van der Waals interactions in these regions. [87]. The hydrophobic portions of InhA inhibitors contribute to their antimycobacterial activity by binding to the enzyme and enhancing lipophilicity for better membrane penetration [88]. The interactions between various ligands and the InhA enzyme provide opportunities for designing new inhibitors that can bind in this region.

### 6.2 Transmembrane Transport Protein Large (MmpL3)

*Mycobacterium tuberculosis* has a thick and waxy cell envelope with two membranes, including mycolates in the outer membrane [89]. Mycobacterial membrane protein large (MmpL) proteins are a type of lipid-transporting protein that are part of the efflux pump resistance nodulation cell division (RND) superfamily. They have specific lipid substrate transport functions. Mycobacterial membrane small proteins (MmpS) are regulatory proteins that work with MmpL proteins and play a role in virulence. MmpL transporters are potential targets for developing anti-tuberculosis drugs [90]. The *Mtb* genome encodes 13 MmpL proteins, with MmpL3 being involved in mycobacterial outer membrane biosynthesis [91]. MmpL3 is responsible for transporting mycolic acids in the form of trehalose monomycolate (TMM) [92], a precursor of trehalose dimycolate (TDM), and mycolates bound to arabinogalactan in the mycomembrane. Compounds that inhibit MmpL3 have been discovered, leading to the disruption of mycolic acid synthesis [93]. These inhibitors reduce TDM and mycolyl arabinogalactan synthesis while increasing TMM levels [94]. MmpL transporters play a crucial role in mycobacterial physiology and pathogenesis by transporting fatty acids and lipid components to the cell wall. Among the 13 *M. tuberculosis* MmpLs, MmpL3 is the only one known to export trehalose monomycolate (TMMs) [95]. MmpL3 is essential for the survival of *M. tuberculosis* and is considered a promising target for the development of new anti-tuberculosis drugs.

### 6.3 Decaprenylphospho-β-d-ribofuranose 2-oxidase (DprE1)

New findings on mycobacterial targets related to the assembly mechanisms of the distinctive *Mtb* cell wall are still being uncovered [96]. One of the recently highlighted potential bacterial targets is decaprenylphospho-β-D-ribofuranose 2-oxidase (DprE1), a periplasmic protein crucial for the biosynthesis of the mycobacterial cell wall [97]. DprE1, encoded by the gene dprE1 (rv3790), is an essential enzyme in *M. tuberculosis* that catalyses the conversion of decaprenyl-phospho-ribose (DPR) to decaprenyl-phospho-arabinose (DPA), crucial precursors for cell wall components [98]. Working with DprE2, DprE1 facilitates this process through a two-step epimerization reaction [96]. In the initial stage, DprE1 converts DPR to an intermediate decaprenyl-phospho-2′-keto-D-arabinose (DPX) by oxidizing it and reducing the FAD cofactor to FADH2 [99]. Following this, DPX is further reduced to DPA by DprE2, which is dependent on NADH. Both DprE1 and DprE2 are crucial for the viability and proliferation of *M*. *tuberculosis* [100]. Inhibition of DprE1 disrupts cell wall synthesis and leads to bacterial cell death. Therefore, targeting DprE1 with novel ligands can potentially lead to the development of new and more effective drugs for the treatment of tuberculosis.

## 7 *IN SILICO* STUDIES ON THE POTENTIAL INHIBITORY EFFECTS OF FLAVONOIDS ON *MTB* PROTEINS

Numerous drug discovery studies are ongoing to address drug resistance issues, particularly in tuberculosis [101]. Researchers have identified several *Mtb* proteins as potential targets for flavonoid inhibitors, including enzymes involved in essential metabolic pathways and proteins that play a role in the bacteria's ability to evade the host immune response. By using computational modelling techniques, researchers can predict how flavonoids may interact with these proteins, potentially inhibiting their function and disrupting the growth and survival of *Mtb* [102]. Molecular docking is a valuable tool for predicting the activity of drugs against specific target proteins. In a study by Davis *et al* [103], taxifolin flavanonols were found to be potential dual inhibitors of *Mtb* DNA gyrase and aminoacyl-t-RNA synthetase enzymes, crucial for bacterial DNA replication, transcription, and translation. Among the eight flavonoid compounds tested, taxifolin showed the best docking result with a glide score of -8.22 kcal/mol when binding to these target proteins. Molecular dynamic simulations further confirmed the stability of taxifolin at the binding sites of both enzymes throughout the simulation period. These studies can help identify potential flavonoids that may have anti-tubercular activity by targeting specific proteins involved in the survival and replication of *Mtb*. By analysing the binding affinity and interactions between flavonoids and these proteins, researchers can prioritize compounds for further experimental validation and drug development. Additionally, *in silico* studies can provide insights into the mechanisms of action of flavonoids against *Mtb*, helping to optimize their therapeutic potential.

Flavonoids have a versatile chemical structure that can be modified to enhance their properties and the interest in studying these compounds has increased due to their potential health benefits, as evidenced by the French paradox, where populations in the Mediterranean showed a low rate of cardiovascular mortality despite consuming red wine and high levels of saturated fat [37]. The interest in studying these compounds has increased due to their potential health benefits, as evidenced by the French paradox, where populations in the Mediterranean showed a low rate of cardiovascular mortality despite consuming red wine and high levels of saturated fat [104].

## 8 FLAVONOIDS AGAINST NONTUBERCULOUS MYCOBACTERIA

The genus *Mycobacterium* includes a wide range of species, with the most pathogenic being the *Mycobacterium tuberculosis* complex (*Mtb* C) and *Mycobacterium leprae*. The other species are known as nontuberculous mycobacteria (*NTM*) [105, 106]. The species within the NTM group are widespread and diverse, found in various natural environments as well as human habitats, including hospitals [107]. NTM can cause a variety of infections in humans, including lung infections, skin infections, and infections in other parts of the body. These infections are typically not contagious and are usually acquired from the environment [108]. *M. avium* complex is the most common and recurring pathogen among non-tuberculous mycobacteria, causing both pulmonary and extrapulmonary infections. Other significant pulmonary pathogens are responsible for skin and cutaneous tissue infections [109]. *Mycobacterium abscessus, M. fortuitum, M. chelonae,* and *M. chimaera* are the causative agents responsible for the majority of soft tissue infections [110]. The cell wall structures of *M. abscessus* vary depending on the presence or absence of glycopeptidolipids (GPL) [111]. Similarly, other *NTM* species have exhibited structural differences. The colony morphology and arrangement of GPL in *M. abscessus* play a crucial role in interactions with the host, influencing biofilm development and intracellular survival, ultimately impacting disease presentation and clinical outcomes [112]. NTM typically enter the host through direct invasion, such as trauma, iatrogenic acquisition, or post-surgical infections [113]. In immunocompromised individuals, these bacteria can invade soft tissues and skin during systemic dissemination [114].

Flavonoids have a long history of being used to treat a variety of human illnesses. They have shown potential in inhibiting the growth of NTM through various mechanisms such as disrupting cell wall formation, biofilm formation, bacterial DNA synthesis, and efflux pumping systems. Combining flavonoids with antimycobacterial agents could be a more effective strategy to address mycobacterial infections and combat microbial resistance.

### 8.1 Inhibition of the Formation of Cell Walls

Flavonoids inhibit bacterial cell wall formation by interfering with the activity of enzymes involved in cell wall synthesis [115]. They can inhibit bacterial growth by disrupting essential cellular processes, such as interfering with microbial adhesions, disrupting cell wall synthesis, or inhibiting transport proteins. This disruption of cell wall synthesis leads to weakened cell walls and ultimately cell death. Additionally, flavonoids can also disrupt the transport of essential nutrients across the cell wall, further compromising the survival of *NTM* [68, 115].

### 8.2 Inhibition of Biofilm Formation

Biofilms protect bacteria against the host immune system, making infections harder to treat. Additionally, they can act as reservoirs for antibiotic-resistant genes, contributing to the proliferation of antibiotic resistance [116]. Research in this area has sparked interest in the potential of flavonoids to improve outcomes in untreatable infections, particularly those caused by antibiotic-resistant bacteria like *NTM*. Several studies have shown that the structure of flavonoids plays a key role in their antibacterial properties, with flavones and flavanones demonstrating anti-NTM activity and the ability to inhibit biofilm formation due to their structural configuration, with hydrophobic compounds on one aromatic ring and hydrogen-bonding groups on another [117]. By incorporating hydrophobic substituents like heterocyclic moieties, flavonoids can effectively kill bacteria in biofilms and work synergistically with antibiotics [118, 119]. Recent studies have shown that certain flavonoid derivatives, such as apigenin, exhibit significant antimycobacterial activity against NTM species by inhibiting biofilm formation. Apigenin, for example, contains a cyclic or aliphatic chain at the 8-C position, enhancing its antimycobacterial properties and preventing biofilm formation [120].

### 8.3 Efflux-mediated Pumping Systems

Flavonoids are natural compounds that have been shown to have antimicrobial properties. In the context of non*-*tuberculous mycobacteria (NTM), flavonoids may exert their antimicrobial effects through efflux-mediated pumping systems. These systems are responsible for pumping out toxic substances from bacterial cells, helping the bacteria to survive in hostile environments [121]. The gradual accumulation of a diverse range of efflux pumps is a key factor in the development of antibiotic resistance in bacteria. These pumps help bacteria avoid the buildup of antibiotics within their cells. Some flavonoids have been found to inhibit these efflux pumps, which can enhance the effectiveness of antibiotics and restore susceptibility to these drugs in some cases [121, 122]. Flavonoids may interfere with these efflux pumps, leading to an accumulation of toxic substances within the bacterial cells and ultimately causing their death. This mechanism of action makes flavonoids potential candidates for the development of novel antimicrobial agents against NTM infections.

### 8.4 Inhibition of Bacterial DNA Replication

Flavonoids, which are known to inhibit topoisomerases, play a role in the antimycobacterial activity. DNA topoisomerase is an essential enzyme for DNA replication and is a primary target for antimycobacterial agents [75]. Flavonoids inhibit bacterial DNA synthesis by interfering with the activity of enzymes involved in DNA replication. Specifically, flavonoids can inhibit the activity of DNA gyrase and topoisomerase IV, which are essential enzymes for bacterial DNA replication [75]. DNA topoisomerases are enzymes that are involved in resolving changes in DNA topology that occur during processes such as transcription, replication, and recombination. These enzymes are essential for managing the supercoiling that arises from the structure of duplex DNA [123]. By inhibiting these enzymes, flavonoids prevent the bacteria from replicating their DNA, ultimately leading to cell death. Additionally, flavonoids can also disrupt the formation of the DNA replication complex, further inhibiting bacterial DNA synthesis. Several molecular docking studies have demonstrated that quercetin can competitively interact with the ATP binding site in the B subunit of DNA topoisomerase, inhibiting DNA topoisomerase and DNA supercoiling [124, 125]. Additionally, quercetin has been found to disrupt the DNA topoisomerase complex by binding to DNA, leading to bacterial DNA degradation [75].

### 8.5 Bacterial Cell Membrane Disruption

Maintaining membrane integrity is crucial for the bacterial plasma membrane, as it is involved in key functions such as respiration, osmoregulation, cellular transport, peptidoglycan biosynthesis, and cross-linking of peptidoglycan. Disrupting the bacterial plasma membrane can lead to changes in cell fluidity and permeability, potentially causing metabolic dysfunction and ultimately leading to bacterial death [126, 127]. Membrane disruption in non-tuberculous mycobacteria (*NTM*) can be caused by various factors, including antibiotics, antimicrobial peptides, and host immune responses. *NTM* are known to have a complex cell wall structure that can make them resistant to certain antimicrobial agents. Disruption of the cell membrane can lead to leakage of cellular contents, loss of membrane integrity, and ultimately cell death [128]. This can be a key mechanism of action for certain antibiotics that target the cell membrane of bacteria. Additionally, host immune responses, such as the production of antimicrobial peptides, can also disrupt the cell membrane of *NTM* and contribute to their clearance from the body.

Flavonoids exhibit antimicrobial activity by interacting with lipid bilayers due to the presence of both polar and non-polar residues in their structure. The non-polar groups can interact with the hydrophobic interior of the membrane, while the polar head groups and hydrophilic residues form hydrogen bonds at the membrane surface [129]. This interaction reduces the fluidity in both hydrophilic and hydrophobic regions of the cellular membrane, leading to antibacterial effects. Catechin, for example, can bind to the lipid bilayer and disrupt the bacterial membrane by generating reactive oxygen species and causing potassium leakage in MRSA at concentrations ranging from 5-15 ppm [130, 131].

## 9 FLAVONOIDS AS A POTENTIAL SOLUTION TO COMBAT DRUG-RESISTANT TUBERCULOSIS STRAINS

The World Health Organisation reports 2024, stated that Tuberculosis (TB) is a preventable and often curable disease. However, in 2023, TB likely regained its status as the leading cause of death from a single infectious agent, surpassing COVID-19, and resulted in nearly double the number of deaths compared to HIV/AIDS. Over 10 million people still contract TB annually, with the number of cases increasing since 2021. Immediate measures are needed to eliminate the global TB epidemic by 2030 (WHO, 2024). The rapid spread of multidrug-resistant (MDR) and extensively drug-resistant (XDR) bacteria worldwide highlights the critical need for new approaches to antibiotic treatment in order to address global infectious diseases like tuberculosis [132].

Mycobacteria are known for their hydrophobic cell envelope and the presence of multidrug efflux pumps (EPs). Putative drug efflux genes and similar pumps have been found *in M. smegmatis, M. aurum, M. bovis,* and *Mtb* [133, 134]. These EPs are just one example of the numerous resistance mechanisms that bacteria have evolved to survive in the presence of chemotherapeutic drugs [122]. These transmembrane proteins function as efficient tools by removing harmful substances from bacterial cells, helping to prevent the buildup of antimicrobial drugs inside the cells. Therefore, targeting efflux pumps could be a promising strategy to combat increasing antibiotic resistance and introduce a novel approach to drug therapy [135, 136].

Despite the various obstacles that need to be overcome, including the risk of resistance development in mycobacteria exposed to subinhibitory concentrations of efflux pump inhibitors (EPI), ensuring comparable pharmacokinetics of adjuvant and antitubercular drugs, and the specificity of EPI for bacterial efflux pumps rather than eukaryotic transporters, integrating an EPI into a treatment regimen may help to revive the efficacy of antibiotics that are losing their effectiveness [137]. It is crucial to highlight that currently, no efflux pump inhibitor has been authorized for clinical application [55].

There has been a growing interest in the discovery of new efflux pump inhibitors from natural sources, such as flavonoids. Several flavonoids have demonstrated the ability to enhance the susceptibility of NTM to isoniazid, with the flavonol myricetin showing the highest activity [138]. Additionally, biochanin A, an isoflavone, showed efflux pump inhibiting activity in *M. smegmatis* similar to verapamil (VP). As a result, it was chosen as a model for developing potent 3-phenylquinolone efflux inhibitors in *M. avium* [138]. Due to the significant challenges presented by multidrug-resistant pathogens, particularly mycobacteria, combining a plant-derived efflux pump inhibitor with an antibiotic may offer enhanced clinical advantages in treating infectious diseases [139]. Flavonoids have shown promise as a group of plant phenolics in this regard.

## CONCLUSION

Despite the identification of numerous potential chemical compounds with anti-mycobacterial properties, addressing multidrug-resistant and extensively drug-resistant *M. tuberculosis* strains remains a significant challenge. Research has focused on understanding the drug resistance mechanisms of Mycobacterium species to pinpoint potential drug targets, such as cell wall synthesis, bacterial DNA and RNA synthesis, and protein synthesis. Flavonoids in plants, such as quercetin, rutin, apigenin, and catechin, exhibit promising antimycobacterial activity against *M. tuberculosis*. Various flavonoids extracted from plants, including new Isoflavonoids from *Rhynchosia precatoria*, have been evaluated for their antimycobacterial properties. Six compounds, namely precatorin A, precatorin B, precatorin C, lupinifolin, and cajanone, have shown the ability to inhibit the growth of *M. tuberculosis* at a minimum inhibitory concentration of 31.25 µg/mL. Further, *in vivo* studies are necessary to assess their potential as an alternative treatment of tuberculosis, especially against different *M. tuberculosis* strains due to escalating drug resistance issues. Ongoing drug discovery efforts are targeting tuberculosis drug resistance, with a focus on mycobacterial targets involved in constructing the unique *Mtb* cell wall. Key enzymes like Decaprenylphospho-β-D-ribofuranose 2-oxidase (DprE1) and Enoyl Acyl Carrier Protein (ACP) Reductase (InhA) play crucial roles in cell wall biosynthesis and mycolic acid biosynthesis, respectively. Investigating interactions between various ligands and the InhA enzyme presents opportunities for designing new inhibitors. The discovery of a novel ligand capable of inhibiting *M. tuberculosis* could lead to more affordable and effective treatments with potentially fewer side effects, benefiting global health by providing accessible options for tuberculosis patients. Continued research and development in this area could result in the identification of highly efficient tuberculosis treatments.

## Figures and Tables

**Fig. (1):**
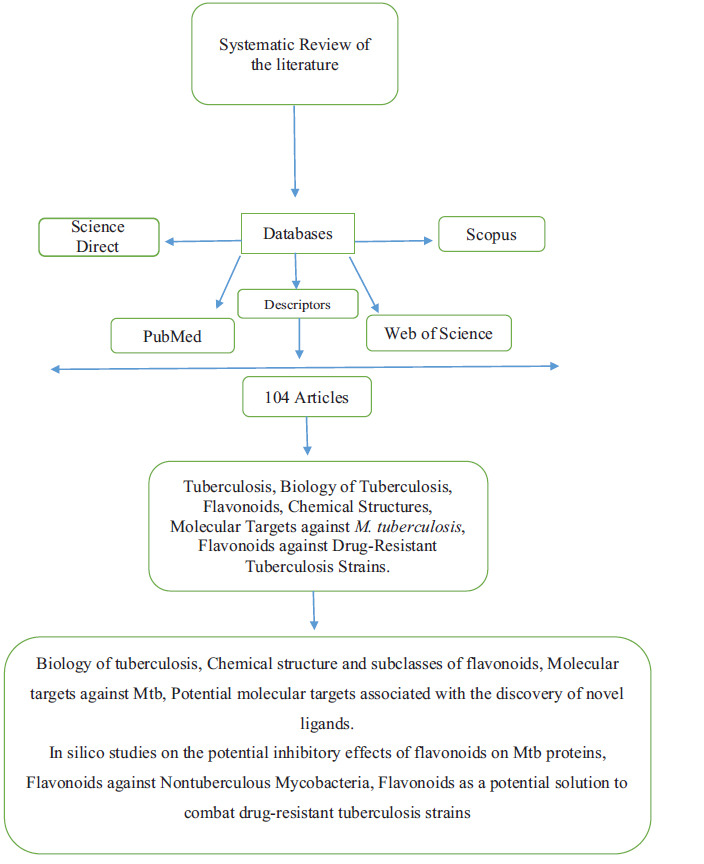
Method of selection of articles included in the review.

**Scheme 6:**
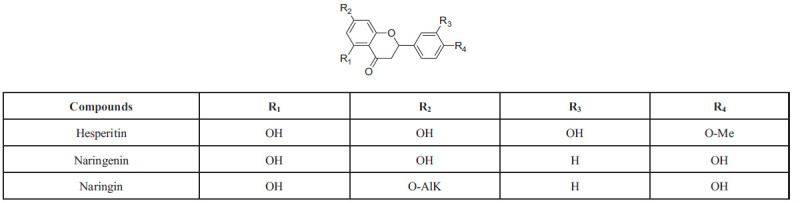
Flavonones: overview of chemical structure, different types, and positions of substitution within the core skeleton.

**Scheme 7:**
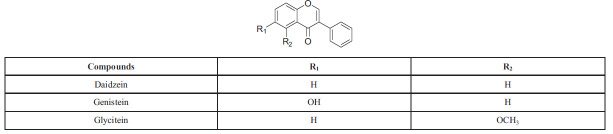
Isoflavonoids: chemical structure, types and substitution positions in the basic skeleton.

**Table 1:** The mechanism of action of certain flavonoids.

**S. No.**	**Compounds**	**Mechanism**						**References**
1	Chrysin-flavone	Inhibitors that effectively block multidrug transporters.						[42]
2	Genistein-Isoflavone	Inhibitors that effectively block multidrug transporters.						[43]
3	Isoliquirtigenin, Butein	Block the activity of fatty acid synthase II, which is involved in the production of mycolic acid.						[44]
4	Quercetin	Bind to the Toprim domain of the B subunit of DNA gyrase, which is essential for the enzyme's function.						[45]
5	Myricetin, Kaempferol, biacalein	Mtb proteasome inhibitors.						[46]
6	Fluorquinolone-flavonoid (hybrid)	Inhibitory effects on efflux pumps.						[24]
7	Baicalin	Prevent the phosphorylation of NFκB.						[47]
8	Epigallocatechin gallate	Blocking the activity of Mtb enoylacyl reductase.						[48]
9	Fisetin	Block the synthesis of fatty acids and mycolic acids in mycobacteria.						[44]

## LIST OF ABBREVIATIONS

TB: Tuberculosis
MDR: Multidrug-Resistant
XDR: Extensively Drug-Resistant
ACP: Acyl Carrier Protein
